# Adherence to standard precaution measures between pre-and in-hospital emergency nursing professionals in a northeast county

**DOI:** 10.5327/Z1679443520190390

**Published:** 2019-12-01

**Authors:** Aline Maria Veras Mendes, Magda Milleyde de Sousa Lima, Dariane Veríssimo de Araújo, Izabelle Mont’Alverne Napoleão Albuquerque, Luciana Maria Montenegro Santiago, Lívia Moreira Barros

**Affiliations:** 1 Nursing, Sate University of the Acaraú Valley - Sobral (CE), Brazil. Universidade Estadual do Vale do Acaraú Nursing Sate University of the Acaraú Valley Brazil

**Keywords:** occupational risks, health services, nursing, team

## Abstract

**Introduction:**

Health professionals are constantly exposed to occupational accidents. In this context, standard precautionary measures (PP) were established, designing biosafety standards at all stages of care, but several factors influence low adherence.

**Objective:**

To evaluate the adherence of nursing professionals working in emergency services to standard precautionary measures.

**Method:**

This is an exploratory study with quantitative approach developed in northern Ceará from June to July 2018. Data were collected from the instruments: clinical-epidemiological data and the scale of adherence to PPS. Statistical analysis was performed using SPSS version 22.0 software.

**Results:**

Of the 86 interviewees, 20.93% were linked to SAMU and 79.07% to hospital emergency trauma-orthopedics, distributed among 79.07% nursing technicians and 20.93% nurses, being 45.34% gender male and 54.65% female. Data analysis identified a statistically significant difference when comparing intermediate and high adherence levels in the items: follow standard precautions with all patients regardless of diagnosis (p=0.05); wear a protective apron when dealing with secretions or blood (p=0.000); wear protective glasses when dealing with blood or secretions (p=0.000); wear a disposable mask (p=0.001) and immediately wipe off any blood or other secretions (p=0.002).

**Conclusions:**

Non-compliance with PP is due to an association of interrelated factors that refers to the units personal, organizational and structural profile. Therefore, the development of actions to promote guidance to professionals is indispensable.

## RESUMO |

**Introdução:**

Os profissionais de saúde estão constantemente expostos à ocorrência de acidentes ocupacionais. Nesse contexto, foram estabelecidas as medidas de precaução padrão (PP) concebendo normas de biossegurança em todas as etapas da assistência, contudo diversos fatores influenciam a baixa adesão.

**Objetivo:**

Avaliar a adesão dos profissionais de enfermagem que atuam nos serviços de urgência e emergência às medidas de precaução padrão.

**Método:**

Trata-se de estudo exploratório com abordagem quantitativa desenvolvido no norte do Ceará no período de junho a julho de 2018. Os dados foram coletados mediante os instrumentos: dados clínico-epidemiológicos e escala de adesão às PP. A análise estatística foi feita por meio do *software* Statistical Package for the Social Sciences (SPSS) versão 22.0.

**Resultados:**

Dos 86 entrevistados, 20,93% eram vinculados ao Serviço de Atendimento Móvel de Urgência (SAMU) e 79,07% à emergência hospitalar de traumato-ortopedia, distribuídos entre 79,07% técnicos de enfermagem e 20,93% enfermeiros, sendo 45,35% do gênero masculino e 54,65% do feminino. A análise dos dados identificou diferenças estatisticamente significantes ao comparar o nível de adesão intermediário e alto nos itens: seguir as PP com todos os pacientes seja qual for o diagnóstico (p=0,05); usar avental protetor ao lidar com secreções ou sangue (p=0,000); usar óculos protetores ao lidar com sangue ou secreções (p=0,000); usar máscara descartável (p=0,001); e limpar imediatamente todo o derramamento de sangue ou de outras secreções (p=0,002).

**Conclusões:**

Constata-se que a não conformidade às PP se deve a uma associação de fatores inter-relacionados que remetem ao perfil pessoal, organizacional e estrutural da unidade. Portanto, torna-se indispensável o desenvolvimento de ações para promover orientação aos profissionais.

## INTRODUCTION

Hospital workers are constantly exposed to different hazards, particularly those allocated to emergency departments as a function of their direct contact with trauma victims and other patients with several conditions. In addition, exhausting working hours increase the odds of work accidents, with the consequent impact on their work and personal life^1^. Few studies, however, analyzed work accidents among prehospital emergency care providers. Those which described hazards to which these workers are exposed evidence a need for effective adherence to standard precautions^2^.

Occupational accidents among health care workers are those which occur during care delivery and correspond to five categories of hazards: biological, physical, chemical, ergonomic and accidents. Incidents involving biological materials are the most frequent among nursing professionals given their frequent exposure to body fluids during the performance of their tasks.

In 1996 the Centers for Disease Control and Prevention established standard precautions including biosafety measures for all stages of care delivery and equipment handling regardless of the patient’s diagnosis, which include handwashing and personal protective equipment (PPE) as e.g. gloves, gowns, masks and safety goggles^3^.

Low adherence to such measures usually does not result from one single reason, but to the coexistence of many, such as poor availability of equipment, work overload, precarious physical infrastructure, difficult adjustment to wearing PPE, lack of time, self-confidence and time in the job^4^. As a result, the rate of accidents is still high, with the consequent impact on the workers’ lives, as well as financial losses due to the cost of prophylaxis, mostly derived from neglect or improper use of available PPE. Therefore staff’s awareness on use of PPE, preventive immunization and continuing education needs to be raised to accomplish the goals of high-quality and risk-free care delivery.

Better technology and new protocols do not suffice to safeguard the health of workers, but investment in education is also required^5^. Within this context one may ask: what is the level of adherence of prehospital and emergency care workers to standard precautions? What are the factors that hinder adherence?

The overall aim of the present study is to contribute to the management of emergency and urgent care services by investigating the level of adherence to standard precautions and understanding better the working conditions to which workers are exposed to thus provide useful information for planning accident prevention strategies. For this purpose, we analyzed the adherence of nursing professionals allocated to emergency and urgent care services to standard precautions.

## METHODS

The present exploratory and quantitative study was performed in June and July 2018 at a trauma referral hospital and Mobile Urgent Care Service (MUCS) in the Sobral macroregion, in the north of the state of Ceará, Brazil.

The study population comprised all 107 prehospital and in-hospital nursing staff members. Inclusion criteria were: nursing professionals allocated to the hospital emergency department or MUCS. Exclusion criteria were: workers on sick or maternity leave or vacation and those with less than 3 months in the job. Following application of the inclusion and exclusion criteria, the sample decreased to 86 participants, which represents a loss of 19.7% (n=21). Five workers refused participation, eight had changed their shift schedule on the day of data collection, four were on vacation, two had been transferred to another department and further two had been less than 3 months in the job.

Data collection was performed in the workplace. We first informed the participants about the study aims and requested them to sign an informed consent form. Next we delivered the data collection instrument, which comprised two sections, the first for clinical-epidemiological and occupational data-sex, age, marital status, occupational category, time in the job, number of jobs, hepatitis B vaccination status, occupational diseases, factors hindering adherence to standard precautions, most frequent accidents and training provided in the workplace.

The second section consisted of a questionnaire on adherence to standard precautions translated and validated for use in Brazil by Brevidelli e Cianciarullo^6^. This scale comprises 13 items which are responded on a Likert scale with the following response options: 1=never, 2=seldom, 3=sometimes, 4=often and 5=always; higher scores indicate better adherence. The results were analyzed through calculation of the mean score on each item and categorized as: high ≥4.5, intermediate 3.5-4.49 or low ≤3^7^^,^^8^.

The data were tabulated using software Excel 2016 and expressed as absolute and relative frequencies. Analysis was performed with Statistical Package for the Social Sciences (SPSS) version 22.0 and consisted in descriptive statistics (frequencies, measures of central tendency and dispersion). We first categorized the participants’s level of adherence to standard precautions (high, intermediate or low) and investigated differences between proportions by means of Pearson’s χ^2^ test and between continuous variables with the Mann-Whitney test. The latter was also used to compare the mean score on each item between the participants with high and intermediate level of adherence to standard precautions.

In compliance with the bioethical principles of autonomy, beneficence, non-maleficence and justice, described in the National Health Council Resolution no. 466/12 on research involving human beings^9^, the present study was submitted to and approved by the research ethics committee of State University of Aracaú Valley, ruling no. 2,738, 986, Certificate of Presentation for Ethical Appraisal no. 84213318.0.0000.5053.

## RESULTS

About 20.93% (n=18) of the participants worked at MUCS and 79.07% (n=68) at the analyzed hospital and were nursing technicians (79.07%, n=68) and nurses (20.93%, n=18), 45.35% (n=39) males and 54.65% (n=47) females. To achieve a better understanding of the collected data, we divided the participants in two groups, to wit, high or intermediate adherence to standard precautions. As Table 1 shows, most participants in both groups were female: intermediate- 53.6% (n=37) vs. 46.4% (n=32); high-58.8% (n=10) vs. 41.2% (n=7).

**Table 1.** Profile of nursing professionals at Mobile Urgent Care Service (MUCS) and hospital trauma department, Sobral, Ceará, Brazil, 2018 (n=86).

Variables	Level of adherence				p**
	Intermediate		High		
	N	%	N	%	
Sex					0.615
Male	32	46.4	7	41.2	
Female	37	53.6	10	58.8	
Mean age (standard deviation)	32.46 (±7.70)		31.94 (±5.05)		0.712*
Marital status					0.848
Single	45	65.2	9	52.9	
Married	22	31.9	7	41.2	
Divorced	1	1.4	1	5.9	
Widowed	1	1.4	0		
Mean years in the job (standard deviation)	6.75 (5.19)		5.65 (2.14)		0.978*
Working hours	53.25 (19.69)		44.82 (15.33)		0.035*
Occupational group					0.011
Nurse	10	14.5	8	47.1	
Nursing technician	59	85.5	9	52.9	
Department					0.509
MUCS	16	23.2	2	11.8	
HHMS	53	76.8	15	88.2	
More than one job					0.298
Yes	39	56.5	7	41.2	
No	30	43.5	10	58.8	
Hepatitis vaccination					0.287
Yes	67	97.1	15	88.2	
No	2	2.9	2	11.8	
Participation in training					0.653
Yes	51	73.9	14	82.4	
No	18	26.1	3	17.6	
Hazards involved in accidents					0.940
Biological	5	7.2	-	-	
Chemical	-	-	-	-	
Sharps	53	76.8	14	82.4	
Physical	6	8.7	2	11.8	
Other	5	7.2	1	5.9	

HHMS: Holy House of Mercy of Sobral; *Mann-Whitney test; **Pearson’s χ^2^ test.

Most common work accidents, in decreasing order, were: intermediate adherence group-sharps injuries (76.8%, n=53), incidents involving physical hazards (8.7%, n=6), biological materials (7.2%, n=5) and other (7.2%. n=5); high adherence group-sharps injuries (82.4%, n=14), physical hazards (11.8%, n=2) and other (5.9%, n=1). Accidents involving chemical hazards were not recorded for either group.

Table 2 describes factors hindering adherence to standard precautions. The most frequent reasons depended on personal aspects, namely, lack of awareness, followed by lack of preparation, time and training. The participants also mentioned structural aspects, such as lack of equipment. Organizational and management aspects included job demands, work overload and lack of incentive.

**Table 2.** Subjective factors participants mentioned as hindering adherence to standard precautions at Mobile Urgent Care Service (MUCS) and hospital trauma department, Sobral, Ceará, Brazil, 2018 (n=86).

	Number of records
Lack of awareness	32
Lack of equipment	31
Work overload	19
Lack of preparation and training	16
Lack of incentive	9
Job demands	7
Lack of time	3

The results relative to the questionnaire on adherence to standard precautions are described in Table 3. Analysis evidenced statistically significant difference between groups intermediate and high adherence relative to the following items: adherence to precautions while providing care to any patient regardless of diagnosis (p=0.05), wearing a gown when dealing with blood or secretions (p=0.000), wearing safety goggles when dealing with blood or other secretions (p=0.000), wearing a disposable face mask (p=0.001) and immediately cleaning spilled blood or other body fluids (p=0.002).

**Table 3.** Scores on the scale of adherence to standard precautions relative to nursing professionals at Mobile Urgent Care Service (MUCS) and hospital trauma department, Sobral, Ceará, Brazil, 2018 (n=86).

Items	Level of adherence		P*
	Intermediate	High	
	Mean (SD)	Mean (SD)	
1. Proper disposal of sharps	4.99 (0.120)	5 (0.00)	0.878
2. All patients approached as if contaminated with HIV	4.03 (1.175)	4.59 (0.712)	0.78
3. Compliance with all standard precautions with all patients regardless of diagnosis	4.46 (0.778)	4.94 (0.243)	0.05
4. Handwashing after removing gloves	4.51 (0.720)	4.94 (0.243)	0.039
5. Wearing a gown when dealing with blood or secretions	3.43 (1.206)	5 (0.00)	0.000
6. Wearing disposable gloves when dealing with blood or secretions	4.86 (0.601)	5 (0.00)	0.583
7. Wearing safety goggles when dealing with blood or secretions	2.86 (1.287)	4.59 (0.618)	0.000
8. Wearing disposable face mask	4.42 (0.775)	5 (0.00)	0.001
9. Immediate cleaning spilled blood or other body fluids	4.09 (1.054)	4.88 (0.485)	0.002
10. Careful handling of scalpels and other sharps	4.77 (0.667)	5 (0.00)	0.234
11. Needle recapping during vein puncture	1.72 (0.889)	1.76 (1.20)	0.661
12. Wearing gloves during vein puncture	4.77 (0.546)	5 (0.00)	0.06
13. All materials in contact with patients are considered as being contaminated	4.68 (0.653)	4.82 (0.393)	0.765
Mean (global)	4.12	4.65	0.000

SD: standard deviation; *Mann-Whitney test for intergroup comparison; HIV: human immunodeficiency virus.

## DISCUSSION

The results evidence a predominance of females, which corresponds to the overall profile of the nursing profession in Brazil. Indeed, according to the Federal Nursing Council, 84.6% of nursing professionals are female. However, the fact that men currently represent 15% of this occupational group points to an increasing masculinization of the profession since 1990^10^.

The average age of the sample was around 30, i.e. a young population. This finding agrees with those of a study conducted at a MUCS in Minas Gerais, Brazil, in which 77.76% of workers were aged 26 to 35. The predominance of this age range in emergency care services is related to the professional profile required in this field, characterized by demands of physical strength and agility in large part of tasks^4^^,^^11^.

Most participants had 5 to 6 years in the job, which is considered ideal from the perspective of stability, since workers have already been through the period of adjustment to department routines^12^. However, some authors observe that workers with longer time in the job are more resistant to wearing PPE and to adopt care measures as a function of bad habits acquired along their career which favor high-risk behaviors^13^.

We found considerable difference between the number of nursing technicians and nurses, which proportions are established on the basis of several factors related to the intrinsic needs of the various departments. A study performed at the emergency department of a hospital in São Paulo, Brazil, found that as a function of the care provided the staff should be composed of 37% of nurses and 63% of nursing technicians^14^.

Hepatitis B vaccination was complete and up-to-date for 97.1% of the participants with intermediate adherence and 88.2% of those with high adherence. These findings agree with those of a study performed at a hospital in Maceió, Brazil, according to which 86.6% of the sample adhered to vaccination^15^. However, some participants reported to be unfamiliar with the vaccination schedule, which points to lack of awareness on the relevance of immunization for disease prevention. Therefore, health institutions should periodically carry out vaccination campaigns, particularly during epidemics.

Among the most common accidents, sharps injuries stood out, with a mean prevalence of 79.6%. This rate may increase in up to 200% as a function of too long working hours and improper infrastructure. These facts indicate that more management actions are needed to attain desirable levels of safety through risk prevention programs and improved workers’ awareness^16^. This situation agrees with that depicted in a study conducted in Ethiopia, which evidenced a high rate of accidents involving contaminated sharps-which may transmit HIV, B and C hepatitis-among nursing professionals, of 34.5% along one year^17^^,^^18^. Incidents involving other types of hazards were less frequent, and none involving chemicals was reported. While biological hazards were exclusively mentioned by the participants with intermediate adherence to standard precautions, one should bear in mind these hazards are also involved in sharps injuries. Physical and other types of hazards were mentioned, albeit less frequently.

The results reveal resistance to wearing PPE among the participants due to difficulty to adjust to equipment, self-confidence, a too large number of patients, lack of information and training, and poor investment in continuing education and professional training. These findings agree with those of an integrative review of nine studies published from 2010 to 2015, which evidence improper use of PPE associated with lack of workplace safety standard operational and technical protocols, self-confidence and lack of equipment^19^. Risk substantially increases among workers who fail to adopt preventive behaviors during the performance of procedures due to unavailability of courses or to lack of awareness. Providing training is necessary for staff members to be able to detect hazards, improve their skills and prevent adverse events.

Gloves and face masks were the PPE items with the highest levels of adherence. Considering also other studies, the rate of adherence varies from 82 to 100%^20^. Among the participants with intermediate level of adherence, gowns and safety goggles were the least worn items. Some authors believe that reasons for non-compliance are lack of availability, not having developed a habit or difficulty in wearing PPE items^21^.

While proper disposal of sharps exhibited a satisfactory level of adherence, at the same time these agents were the main source of accidents, whence one may conclude that this aspect still represents one of the main problems.

Most participants reported to wash their hands after removing gloves. One of the simplest and least expensive precautions, handwashing should be continually enhanced as a function of its efficacy for infection transmission prevention^22^.

Lack of awareness and of PPE were main reported hindrances to adherence to standard precautions and both contribute to the occurrence of work accidents. These findings agree with those of a study performed at a university hospital in the Federal District, Brazil, which evidenced that lack of knowledge and unavailability of PPE had direct influence on non-adherence to standard precautions^23^. To solve this problem, the authors of a study conducted in Nigeria suggest providing continuing training to workers and ensuring proper availability of the required equipment^24^.

Work overload was also mentioned as hindering adherence to standard precautions. According to Correia et al., working two shifts is a cause of stress and tiredness and thus contributes to the occurrence of sharps injuries in particular ^25^.

Some aspects stood out upon comparing the groups with intermediate and high adherence to standard precautions, to wit: wearing a gown when dealing with blood or secretions, wearing safety goggles when dealing with blood or secretions, wearing a disposable face mask and immediately cleaning spilled blood or other body fluids. A study performed at a public hospital in Rio de Janeiro, Brazil, found that 68% of the nursing staff wore gowns and 69.2% safety goggles and face masks when exposed to blood and secretions, and 93% cleaned spilled blood. These findings help in the development of strategies to enhance adherence to standard precautions^26^. Yet a study conducted in Italy identified gaps in the knowledge on disinfection and sterilization among about 50% of the participants, which contribute to contamination among workers and infection among patients, with high financial costs for both workers and institutions^27^.

Our findings relative to global adherence to standard precautions agree with those of other studies, i.e. in most the level of adherence was intermediate. Ferreira et al.^3^ conducted a study with the nursing staff at the internal medicine department of a teaching hospital in Minas Gerais, Brazil, to investigate the relationship among level of adherence, occupational group and time in the job. The results showed that scores corresponded to category intermediate for 55.5% of 54 participants and that the items with best adherence were proper disposal of sharps, wearing disposable gloves when dealing with blood, careful handling of scalpels and considering all objects in contact with patients as being contaminated^3^. In a study performed in 2016 and 2017 at a university hospital in the south of Brazil, 57.6% of the participants exhibited intermediate scores on adherence to standard precautions, which denotes partial compliance with measures^28^. Contrariwise, a study with 256 nursing professionals at a teaching hospital in São Paulo found that most, 59.4%, exhibited high scores vs. 38.3% with intermediate scores^29^. In turn, the authors of a study performed at a psychiatric hospital in São Paulo evidenced that nursing professionals had appropriate knowledge of hazards intrinsic to their occupation and that proper compliance with standard precautions is influenced by cultural and organizational factors^7^. According to Porto and Marziale, undergraduate training is one of the reasons which account for poor adherence to standard precautions, whence educational actions in the workplace are needed to promote changes of behavior and thus improve the adherence rates^30^.

The limitations of the present study derive from the large number of excluded workers, inadequacy of the inclusion/exclusion criteria and the fact it was performed within the public health system only, which hinders comparison to workers in the private sector and generalizing the results to other regional contexts. Thus, we point to the need for additional studies on adherence to standard precautions among nursing professionals in other health facilities across Brazil to raise the awareness of this occupational group on the use of PPE.

## CONCLUSION

MUCS and trauma department nursing staffs were mainly composed of nursing technicians, female and single workers, aged 30 on average, with 5 to 6 years in the job and adequately vaccinated against hepatitis B.

We found statistically significant difference between participants with intermediate and high level of adherence relative to items: wearing a gown when dealing with blood or secretions, wearing safety goggles when dealing with blood or secretions, wearing a disposable face mask and immediately cleaning spilled blood or other body fluids.

Adhering to standard precautions is essential to safeguard workers, reduces contamination by body fluids containing pathogenic agents and excretions, and helps prevent infection among patients, thus it shortens their length of stay at hospital and decreases the financial costs to health services. Managers play a fundamental role in this regard through the implementation of educational actions to provide orientation, raise awareness on, afford incentives and training to workers on appropriate use of PPE and on the relevance of complying with biosafety guidelines to ensure safe practice. Similarly, also institutions should provide the necessary equipment, hire more workers and establish standard operational procedures for the use of equipment.

Finally, additional studies are necessary with a larger number of workers in clinical, obstetric, psychiatric and cardiac emergency care to increase and compare data on this subject.

## References

[1] Loro MM, Zeitoune RCG, Guido LA, Silveira CR, Silva RM (2016). Desvelando situações de risco no contexto de trabalho da Enfermagem em serviços de urgência e emergência. Esc Anna Nery.

[2] Leite HDCS, de Carvalho MTR, Cariman SLS, Araújo ERM, Silva NC, Carvalho AO (2016). Risco ocupacional entre profissionais de saúde do serviço de atendimento móvel de urgência-samu. Enfermagem em Foco.

[3] Ferreira LA, Peixoto CA, Paiva L, Silva QCG, Rezende MP, Barbosa MH (2017). Adherence to standard precautions in a teaching hospital. Rev Bras Enferm.

[4] Llapa-Rodríguez EO, Silva GG, Lopes D, Campos MPA, Mattos MCT, Otero LM (2018). Measures for the adhesion to biosafety recommendations by the nursing team.

[5] Oliveira RM, Leitão IMTA, Silva LMS, Figueiredo SV, Sampaio RL, Gondim MM (2014). Estratégias para promover segurança do paciente: da identificação dos riscos às práticas baseadas em evidências. Esc Anna Nery.

[6] Brevidelli MM, Cianciarullo TI (2009). Fatores psicossociais e organizacionais relacionados à adesão às precauções padrão. Rev Saúde Pública.

[7] Piai-Morais TH, Orlandi FS, Figueiredo RM (2015). Fatores que influenciam a adesão às precauções-padrão entre profissionais de enfermagem em hospitais psiquiátricos. Rev Esc Enferm USP.

[8] Brevidelli MM (2003). Modelo Explicativo da Adesão as Precauções-padrão: construção e aplicação.

[9] Brasil. Ministério da Saúde (2012). Anuário Estatístico da Previdência Social. Acidentes de trabalho.

[10] Conselho Federal de Enfermagem (2015). Perfil da enfermagem brasileira.

[11] Santana JCB, Sá EBP, Dutra BS, Campos ACV, Melo CL, Barros SG (2015). Perfil dos técnicos em enfermagem de um serviço de atendimento pré-hospitalar. Enfermagem Revista.

[12] Guimarães AT, Vaghetti HH, Lunardi WD, Gomes GC (2011). Gerenciamento do pessoal de enfermagem com estabilidade no emprego: percepção de enfermeiros. Rev Bras Enferm.

[13] Lopes JDSP, de Carvalho TES, Nascimento JF, Alves CAS, Pereira AKP, Rodrigues TS (2017). Características dos acidentes de trabalho com material biológico em profissionais de enfermagem. Rev Eletrônica Acervo Saúde.

[14] Paixão TCR, Campanharo CRV, Lopes MCBT, Okuno MFP, Batista REA (2015). Dimensionamento de enfermagem em sala de emergência de um hospital-escola. Rev Esc Enferm USP.

[15] Verçosa RCM, Monteiro VGN, Ferreira FAS (2014). Acidentes com perfurocortante entre profissionais de enfermagem de um hospital universitário. Rev Enferm UFPE On Line.

[16] Kebede A, Gerensea H (2018). Prevalence of needle stick injury and its associated factors among nurses working in public hospitals of Dessie town, Northeast Ethiopia, 2016. BMC Res Notes.

[17] Antonelli RC, Bellucci JA (2014). Gerenciamento de enfermagem em serviço hospitalar de emergência: revisão integrativa da literatura. Semina Ciênc Biológicas Saúde.

[18] Mideksa L, Feyera T (2014). Needle-Stick Injuries and Contributing Factors among Health care Workers in Public Health Facilities in Jigjiga Zone, Eastern Ethiopia. World J Med Sci.

[19] Barros JSO, Rodrigues AP, Miranda LN, Araújo MAS (2016). A enfermagem e a resistência ao uso dos equipamentos de proteção individual. Cad Graduação.

[20] Wicker S, Stirn AV, Rabenau HF, Gierke L, Wutzler S, Stephan C (2014). Needlestick injuries: causes, preventability and psychological impact. Infection.

[21] Rezende KCAD, Tipple AFV, Siqueira KM, Alves SB, Salgado TA, Pereira MS (2013). Adesão à higienização das mãos e ao uso de equipamentos de proteção pessoal por profissionais de enfermagem na atenção básica em saúde. Ciênc Cuid Saúde.

[22] Ribeiro RP, Vianna LAC (2012). Uso de equipamentos de proteção individual entre trabalhadores das centrais de material e esterilização. Ciênc Cuid Saúde.

[23] Faria LBG, Santos CTB, Faustino AM, Oliveira LMAC, Cruz KCT (2019). Conhecimento e adesão do enfermeiro às precauções padrão em unidades críticas. Texto Contexto Enferm.

[24] Arinze-Onyia SU, Ndu AC, Aguwa EN, Modebe I, Nwamoh UN (2018). Knowledge and practice of standard precautions by health care workers in a tertiary health institution in Enugu, Nigeria. Niger J Clin Practice.

[25] Correia CMA, Souza DF de, Braga ALS, Chrizóstimo MM, Brum AK, Ferreira SCM (2014). Fatores predisponentes e medidas preventivas aos acidentes com materiais perfurocortantes: revisão integrativa. Rev Enferm UFPE On Line.

[26] Floriano DR, Rodrigues LS, Dutra CM, Toffano SEM, Pereira FMV, Chavaglia SRR (2019). Cumprimento às precauções-padrão por profissionais de enfermagem no atendimento de alta complexidade. Esc Anna Nery.

[27] Accardi R, Castaldi S, Marzullo A, Ronchi S, Laquintana D, Lusignani M (2017). Prevention of healthcare associated infections: a descriptive study. Ann Ig.

[28] Cunha QB (2016). Adesão às precauções padrão por trabalhadores de enfermagem de um hospital universitário: estudo de métodos mistos.

[29] Malaguti-Toffano SE, Canini SRMS, Reis RK, Pereira FMV, Felix MAS, Ribeiro PHV (2015). Adesão às precauções-padrão entre profissionais da enfermagem expostos a material biológico. Rev Eletrônica Enfermagem.

[30] Porto JS, Marziale MHP (2016). Motivos e consequências da baixa adesão às precauções padrão pela equipe de enfermagem. Rev Gaúcha Enferm.

